# Drug regulatory affairs under focus: Knowledge and perceptions among pharmacists and pharmacy students

**DOI:** 10.1371/journal.pone.0320599

**Published:** 2025-03-27

**Authors:** Manal A. Ayyash, Razan I. Nassar, Raghad Al-Muhtaseb, Kamel A. Jaber

**Affiliations:** 1 Department of Pharmaceutics and Pharmaceutical Science, Faculty of Pharmacy, Applied Science Private University, Amman, Jordan; 2 Department of Clinical Pharmacy and Therapeutics, Faculty of Pharmacy, Applied Science Private University, Amman, Jordan; 3 Department of Regulatory Affairs, Savvy Pharma, Amman, Jordan; 4 School of Medicine, The University of Jordan, Amman, Jordan; Yarmouk University, JORDAN

## Abstract

**Objectives:**

Pharmaceutical product development, registration, and post-marketing surveillance are the main concerns of the Regulatory Affairs (RA) profession in regulated pharmaceutical industries. The RA assumes a pivotal role in ensuring product quality, patient safety, and drug efficacy and constitutes a vital part of the pharmaceutical industry. The current study aims to evaluate the knowledge and understanding of the concept of RA and Drug Registration (DR) among pharmacists and pharmacy students in Jordan.

**Methods:**

A cross-sectional study was conducted targeting pharmacists in different sectors and pharmacy students to assess knowledge and perceptions of RA and DR in Jordan. The participants were invited to participate by sending the survey link through social media platforms (WhatsApp).

**Results:**

A total of 411 participants completed the study survey. Among the study participants, 193 were pharmacists (47.0%), while the rest were pharmacy students. The majority indicated that they had never taken a course related to RA during their undergraduate studies (77.4%). About half of the participants lacked awareness of RA personnel responsibilities with most of the participants agreeing that workshops, lectures, and training are required.

**Interpretation of results and conclusion:**

This study aims to elevate Jordanian pharmacists’ awareness by focusing the efforts on young pharmacists and pharmacy students. This can be achieved by implementing RA courses within the pharmacy curricula and upholding the role of the regulatory bodies in Jordan.

## Introduction

The pharma industry is one of the regulated industries globally. It is expected to adhere to the guidelines and regulations imposed by the regulatory authorities worldwide to ensure the safety, efficacy, and quality of pharmaceutical products. Regulatory Affairs (RA) is a profession in regulated pharmaceutical industries that covers a wide range of activities including pharmaceutical product development, registration, and post-marketing surveillance [1]. RA assumes a pivotal role in upholding global public health for pharmaceutical companies, ensuring product quality that directly translates into patient safety and drug efficacy [2]. Governments of various countries have developed regulations for pharmaceutical products, cosmetic products, medical devices, and complementary medicines by controlling the safety and efficacy of products [3]. The RA stands as a vital subject, serving as the essential link between pharmaceutical companies and regulatory authorities across the world [4,5]. This intricate field encompasses the meticulous preparation of pharmaceutical dossiers by professionals for submission to health authorities such as the United States Food and Drug Administration (USFDA) and the European Medicines Agency (EMA) [6].

These dossiers play an important role in obtaining regulatory approval from authorities in respective countries where a licensed product necessitates registration or approval for its manufacturing, marketing, use, distribution, or sale.

**Fig 1:**
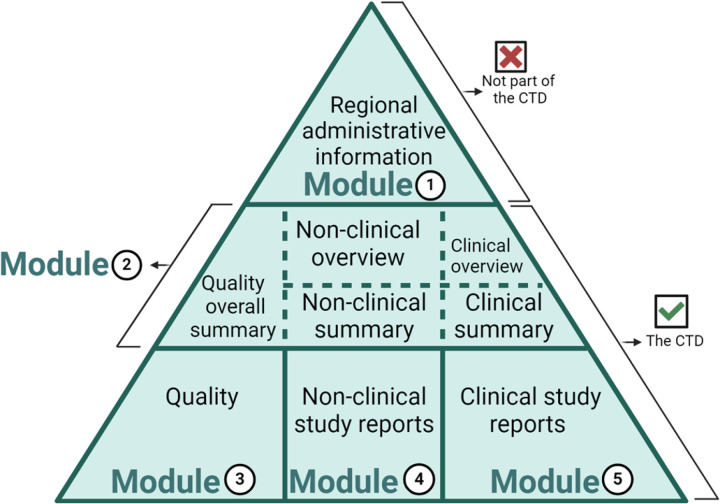
The Common Technical Document (CTD) modules.

The imperative preparation of a dossier adheres to internationally accepted formats such as Common Technical Dossier (CTD) and an electronic Common Technical Dossier (e-CTD), optimizing the submission and registration process for a single drug product across diverse countries [6]. This approach not only minimizes time and effort but also underscores the substantial role of the International Council for Harmonization (ICH) of Technical Requirements for Pharmaceuticals for Human Use in standardizing the process. The ICH’s contribution is manifested in the introduction of the CTD with its five modules (Fig 1), each holding significant information on the quality, safety, efficacy, and toxicity of the drug [7].

The CTD format is a set of specifications for application documents needed for Drug Registration (DR). It was developed by three regulatory agencies, USFDA, Ministry of Health, Labour and Welfare in Japan (MHLW), and EMA [8].

### Roles of RA professionals in pharmaceutical companies

RA professionals play a key role as intermediaries between pharmaceutical companies and health authorities, striving to ensure that high-standard products enter the market [5]. Their provision of strategic and technical counsel throughout a product’s development significantly contributes to a company’s success, both in commercial and scientific terms. In the current competitive landscape, the expeditious market entry of products emerges as a linchpin for a company’s triumph. They are responsible to prepare and submit product registration applications to regulatory authorities with scientific data, manufacturing information, and clinical trial data. RA experts monitor regulatory compliance and ensure that all regulatory submissions, filings, and responses are completed accurately and on time [1]. They work with teams, including research and development (R&D), clinical development, marketing, and legal teams, to ensure alignment between regulatory strategies and business goals [1]. Moreover, RA professionals often need to navigate complex regulatory challenges and find solutions to any unexpected regulatory issues that arise during product development, carrying out all the successive negotiations seeking registration and maintaining marketing authorization for the product [9]. RA professionals adhere to the principles of pharmacovigilance (PV) which is defined by the World Health Organization (WHO) as the “science and activities related to the detection, assessment, understanding, and prevention of adverse effects or any other possible drug-related problems” [10], and commit to follow up, monitor, and report any adverse effects that appear after using the company’s product, and ensure that any necessary corrective measures and actions are taken. This is achieved by submitting Periodic Safety Update Reports (PSUR) which are defined as “The pharmacovigilance documents intended to provide an evaluation of the risk-benefit balance of a medicinal product at defined time points after its authorisation” [11]. PSUR is implemented by many health authorities such as USFDA and EMA in order to track the safety of the authorized drugs.

The regulated pharmaceutical industry in Jordan has flourished over the years, by witnessing an increase in the number of developed manufacturers, producing pharmaceutical products launched to foreign markets, and capable of competing international products, claiming itself among the leading industries around the world [12].

This improvement in the Jordanian pharmaceutical industry necessitates the availability of qualified, trained, and skilled pharmacists, capable of fulfilling such an important and demanding role. Essentially, to create a generation of educated, qualified, and trained pharmacists in RA sectors, RA, and DR must be implemented within the pharmacy curricula.

In light of the deficiency of literature regarding this topic in Jordan and the region, the current study aims to evaluate the knowledge and understanding of the concept of RA within the sector of pharmacy, including pharmacy students and pharmacists in different settings, such as academia, community pharmacies, and pharmaceutical companies.

## Materials and methods

### Study design, settings, and participants

A descriptive-analytical cross-sectional study was conducted from August 29^th^ to December 31^st^, 2023, to assess the knowledge and perceptions of pharmacists working in different sectors and pharmacy students in Jordan regarding RA and DR. In the current study, an appropriate sample was invited to participate by sending the survey link through social media platforms (Facebook and WhatsApp) and before conducting RA workshops organized by the research team. To be included in the study, the participants had to be pharmacists holding a Bachelor of Pharmacy degree (B.Sc.) or pharmacy students of the same degree, being at least 18 years of age, who currently live in Jordan. However, holders of Doctor of Pharmacy Degree (PharmD) and students of this degree were excluded from the study. Participants were informed about the aim of the study and that filling out the survey would take approximately 10-15 minutes, and that their participation was voluntary.

### Survey instrument development and validation

The research teams developed the first draft of the study’s survey to assess the knowledge and perceptions of pharmacists and pharmacy students’ regarding RA and DR. The face and content validities of the draft were evaluated by a group of experts specialised in clinical pharmacy and the pharmaceutical industry. Experts’ comments were collected, leading to the implementation of a few minor modifications to the survey.

The survey consisted of three sections assessing different topics of interest. The survey was comprised of close-ended, multiple-choice questions, and a 5-point Likert scale. The first section consisted of questions that collected participants’ sociodemographic characteristics. The second section assessed participants’ knowledge about RA and DR using multiple choice questions, and “Yes”, “No” and “I do not know” questions. The last section aimed to evaluate the perception of participants regarding RA and DR using a 5-point Likert scale ranging from strongly agree to strongly disagree.

### Ethical consideration

Ethical approval for the current study was obtained from the Ethics Committee provided institutional Review Board (IRB) at Applied Science Private University (Approval number: 2023-PHA-33). The board approved the study conductance and the informed consent procedure. The participants were informed their participation was voluntary, no identifying personal information would be required, their anonymity would be maintained, and all data would be dealt with confidentially, not to be shared or used for purposes outside the scope of the study. After being informed of the study objectives and in order to proceed with filling out the questionnaire, participants who decided to continue were asked to provide their electronic informed consent by clicking on the “*Agree*” button, whereas those who refused to participate could click the “*Disagree*” button.

### Sample size

Using the Epi Info program, the minimum representative sample size was calculated with a 95% confidence level, 50% expected frequency, 5% acceptable margin of error, and a design effect of 1.0. Three hundred and eighty-four participants were the minimum required number of participants. A convenience sampling method was used, as participants were recruited through social media platforms (Facebook and WhatsApp).

### Statistical analysis

Data was analyzed using the statistical package for social science (SPSS) version 27 (SPSS Inc., Chicago, IL, USA). Frequency and percentage were used to represent categorical variables, whereas mean and standard deviation were used to represent continuous variables.

Multiple linear regression was performed to screen for the independent variables affecting the knowledge scores of the goals of RA, the roles of RA professionals, and the general knowledge regarding RA and DR. Simple linear regression was performed first, considering the independent variable eligible to be entered into the multiple linear regression after having a p-value less than 0.25. Subsequently, in the multiple linear regression, any variable with a p-value of 0.05 or less was considered statistically significant. All independent variables were selected after examining their independence, ensuring a tolerance value of < 0.2, and a variance inflation factor (VIF) of < 5 to confirm the absence of multicollinearity.

Three items were added to the goals of RA professionals, leading to the computation of a score on a scale of -3 to 3. Five items were added to the roles of RA professionals, leading to the computation of a score on a scale of -5 to 5. Six items were added to the general knowledge regarding RA and DR, resulting in a score range of -6 to 6. The scoring system assigned a value of + 1 for a correct response, 0 for an “*I do not know*” response, and -1 for an incorrect answer.

## Results

A total of 411 participants completed the study’s survey. More than half of the participants fell within the 18 to 23 age range (51.6%), and 84.9% were females. Among the study’s participants, 193 were pharmacists (47.0%), while the remaining individuals were pharmacy students. Regarding pharmacists’ jobs, 26.5% reported working in a pharmacy, 11.4% in the academic sector, 4.6% in a drug store, and 4.4% in the industry. About one-fifth of the study participants had obtained their bachelor’s degree in pharmacy before 1-3 years, followed by 12.4% who got it before 4-10 years.

The majority of the study’s participants had a Jordanian nationality (82.0%), likewise, the majority were living in the capital, Amman (77.4%). More than 97.0% of the participants reported that they have studied or are currently studying in Jordan, with more than 70.0% studying or having studied in a private university.

Regarding RA, 77.4% indicated that they had never taken a course related to RA during their undergraduate studies. Furthermore, 70.8% of the participants reported that they had never attended a workshop on topics related to RA and DR (Table 1).

**Table 1:** Demographic characteristics of the study participants (n = 411).

Parameters	n (%)
**Age**	
18-23 years 24-30 years 31-40 years More than 40 years	212 (51.6) 112 (27.3) 40 (9.7) 47 (11.4)
**Gender**	
Male Female	62 (15.1) 349 (84.9)
**Are you a pharmacist or a pharmacy student?**	
Pharmacy students (first or second academic year) Pharmacy student (third, fourth, or fifth academic year) Pharmacists	20 (4.9) 198 (48.2) 193 (46.9)
**Which of the following describes your job?**	
Academia Drug store Industry Pharmacy I am still a student	47 (11.4) 19 (4.6) 18 (4.4) 109 (26.5) 218 (53.0)
**How long has it been since you got your pharmacy degree (BSc in pharmacy)?**	
1-3 years 4-10 years 11-20 years More than 20 years I am still a student	85 (20.6) 50 (12.2) 22 (5.4) 36 (8.8) 218 (53.1)
**Nationality**	
Jordanian Non-Jordanian	337 (82.0) 74 (18.0)
**Place of residence in Jordan**	
Amman Other	318 (77.4) 93 (22.6)
**Where did you study pharmacy?**	
Outside Jordan Jordan	12 (2.9) 399 (97.1)
**Are you studying/ have you studied at public (governmental) or private universities?**	
Private University Public (governmental) University	288 (70.1) 123 (29.9)
**During your undergraduate studies, did you take a course or topic related to RA?**	
No Yes	318 (77.4) 93 (22.6)
**Did you attend any lectures or workshops on RA and DR?**	
No Yes	291 (70.8) 120 (29.2)

Assessing participants’ knowledge regarding the definitions of DR and RA showed that 77.1% (n = 317) were familiar with the definition of DR as “*The process of submitting an application to regulatory authorities seeking approval to market and distribute a drug product*”. In comparison, 78.3% (n = 322) were familiar with the definition of drug RA as “*The overall management of regulatory activities related to drug development, registration, and post-marketing surveillance to ensure that all regulatory requirements are met throughout the drug development process*”.

Evaluating participants’ understanding of the goals of RA revealed that most participants were well-informed about these goals, as indicated by their *“Yes”* responses to the three specific goals (Table 2). The participants’ mean knowledge score concerning the goals of RA was 2.43 out of 3 (SD = 1.05).

**Table 2:** Knowledge regarding the goals of RA professionals among students (n = 218) and pharmacists (n = 193).

Statement	Yes n (%)		I do not know n (%)		No n (%)	
Protection of human health.^a^	342 (83.2)		51 (12.4)		18 (4.4)	
	Students 171 (78.4)	Pharmacists 171 (88.6)	Students 39 (17.9)	Pharmacists 12 (6.2)	Students8 (3.7)	Pharmacists 10 (5.2)
Ensuring the safety, efficacy, and quality of drugs^.^^a^	351 (85.4)		43 (10.5)		17 (4.1)	
	Students 175 (80.3)	Pharmacists 176 (91.2)	Students 31 (14.2)	Pharmacists 12 (6.2)	Students12 (5.5)	Pharmacist 5 (2.6)
Ensuring appropriateness and accuracy of product information.^a^	352 (85.6)		48 (11.7)		11 (2.7)	
	Students 177 (81.2)	Pharmacists 175 (90.7)	Students 34 (15.6)	Pharmacists 14 (7.3)	Students 7 (3.2)	Pharmacist 4 (2.1)

^a^: The correct answer is “Yes”.

Assessing participants’ knowledge regarding the role of RA professionals revealed that the majority possessed good awareness, as the correct answers ranged from 70.8% for “*Providing expertise and regulatory intelligence in translating regulatory requirements into practical workable plans*” to 84.4% for “*Ensure adherence and compliance with all the applicable cGMP, ICH, GCP, GLP guidelines, regulations, and laws*” (Table 3). The participants’ mean knowledge score for the role of RA professionals was 3.49 out of 5 (SD = 1.95).

**Table 3:** Participants’ knowledge regarding the roles of RA professionals (n = 411).

Statement	Yes n (%)		I do not know n (%)		No n (%)	
Act as a liaison with regulatory agencies	317^a^ (77.1)		80 (19.5)		14 (3.4)	
	S 155 (71.1)	P 162 (83.9)	S 55 (25.2)	P 25 (13.0)	S 8 (155)	P 6 (3.1)
Preparation of organized and scientifically valid new drug application and drug master file	305^a^ (74.2)		71 (17.3)		35 (8.5)	
	S 137 (62.8)	P 168 (87.0)	S 56 (25.7)	P 15 (7.8)	S 25 (11.5)	P 10 (5.2)
Ensure adherence and compliance with all the applicable cGMP, ICH, GCP, and GLP guidelines, regulations, and laws	347^a^ (84.4)		52 (12.7)		12 (2.9)	
	S 169 (77.5)	P 178 (92.2)	S 42 (19.3)	P 10 (5.2)	S 7 (3.2)	P 5 (2.6)
Providing expertise and regulatory intelligence in translating regulatory requirements into practical workable plans	291^a^ (70.8)		91 (22.1)		29 (7.1)	
	S 141 (64.7)	P 150 (77.7)	S 60 (27.5)	P 31 (16.1)	S 17 (7.8)	P 12 (6.2)
Advising the companies on regulatory aspects and climate that would affect their proposed activities	297^a^ (72.3)		82 (20.0)		32 (7.8)	
	S 145 (66.5)	P 152 (78.8)	S 56 (25.7)	P 26 (13.5)	S 17 (7.8)	P 15 (7.8)

^a^: Correct answer.

S: Students (n = 218).

P: Pharmacists (n = 193).

**Fig 2:**
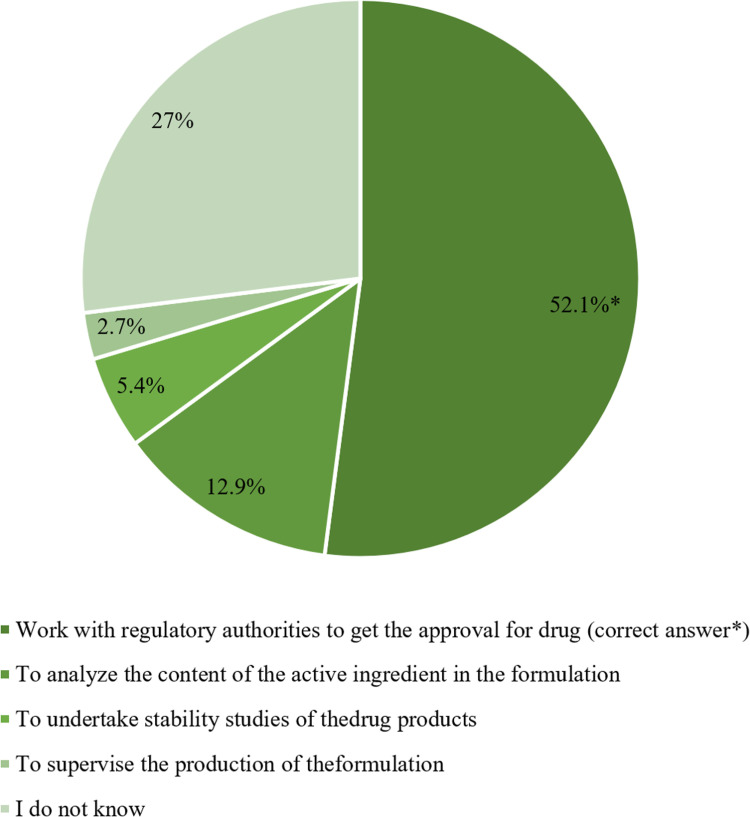
Participants’ awareness of the RA personnel’s responsibility (n =** 411).**

For example, 12.9% (n = 53) thought that their responsibility was to analyze the content of the active ingredient in the formulation (Fig 2).

**Fig 3:**
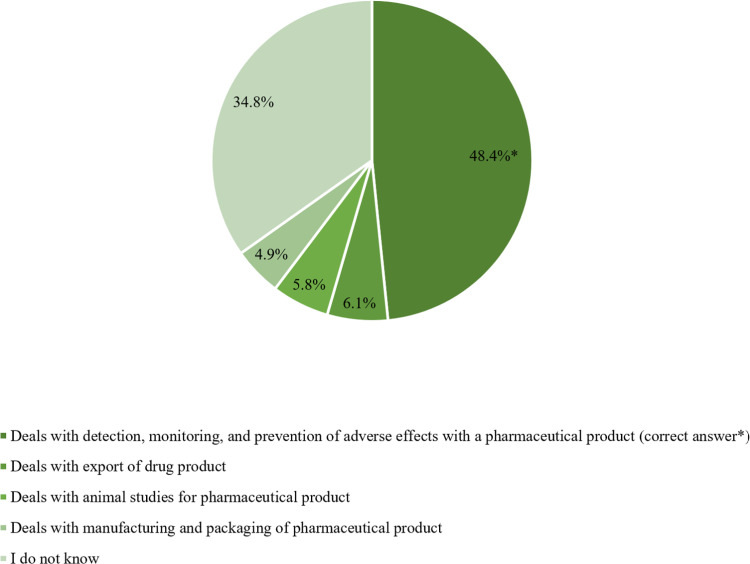
Participants’ awareness of Pharmacovigilance definition (n =** 411).**

Investigating participants’ comprehension of PV indicated that 48.4% (n = 199) were aware of its definition, which involves “*The detection, monitoring, and prevention of adverse effects with a pharmaceutical product*”. Conversely, 34.8% did not know the definition (n = 143), and 16.8% (n = 69) had incorrect perceptions of the PV definition (Fig 3).

Assessing participants’ awareness of the contributors to the ICH initiation revealed that only 24.1% (n = 99) correctly identified the location of the responsible regulatory agencies (Europe, Japan, and the US). On the other hand, 6.8% believed the contributors were Europe, Australia, and the US, 4.4% believed the contributors were Japan, Australia, and the US, and 6.8% thought it was Europe, India, and the US. In addition, 57.9% (n = 238) expressed unawareness by responding with “I do not know”.

Assessing participants’ knowledge regarding the Common Technical Document (CTD) showed that only 88 participants (21.4%) knew that the CTD is divided into five modules. On the other hand, 26.1% of the participants provided incorrect answers, and 52.3% responded with “*I do not know*”.

Participants were asked specifically about CTD module 2 and module 3. Regarding module 2, approximately one-fifth of the participants, accounting for 20.2% (n = 83) recognized its association with all CTD summaries. Conversely, 9.2% believed it pertained to the quality of pharmaceutical products, 7.1% thought it was related to administrative information prescribing information, 3.2% associated it with non-clinical studies, and 60.3% responded with “*I do not know*”. Whereas a lower percentage of participants (16.5%, n = 68) were aware that CTD module 3 is associated with the quality of pharmaceutical products. Conversely, 6.8% believed it was related to all CTD summaries, 6.6% associated it with clinical studies, 4.1% thought it was related to non-clinical studies, 2.9% thought it was related to administrative information prescribing information, and 63.0% responded with “*I do not know*”.

**Fig 4:**
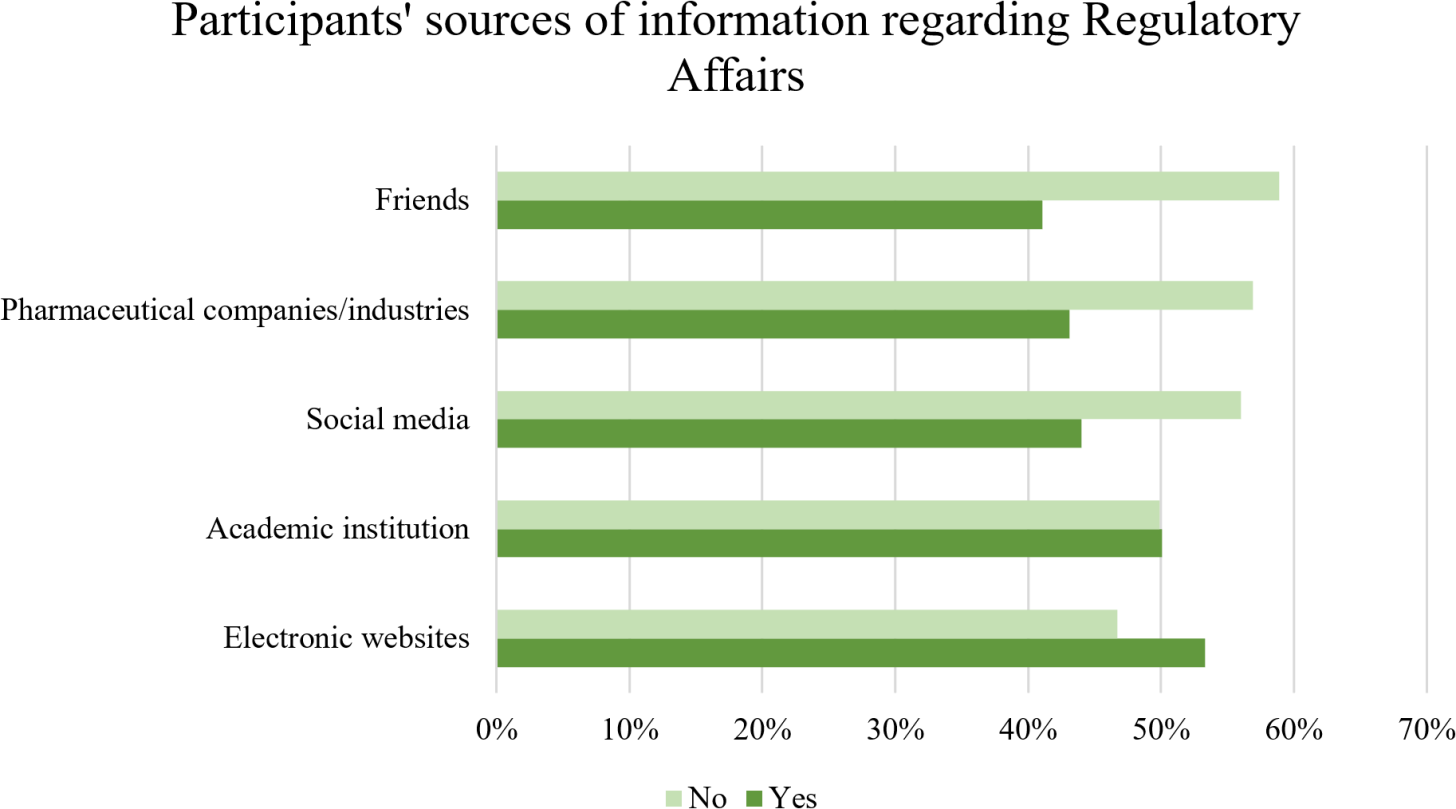
Participants’ sources of information regarding RA (n =** 411).**

Evaluating participants’ sources of information regarding RA (Fig 4) revealed that the primary used source was electronic websites (53.3%), followed by academic institutions (50.1%).

Assessing participants’ general knowledge regarding RA and DR revealed that correct answers ranged between 44.4% for “*Common Technical Document (CTD), is a format set by the ICH*” to 74.2% for “*Active pharmaceutical ingredient (API) is not related to drug substance*” (Table 4). The participants’ mean general knowledge score regarding RA and DR was 3.34 out of 6 (SD = 2.31).

**Table 4:** Participants’ general knowledge regarding RA and DR (n = 411).

Statement	True n (%)		I do not know n (%)		False n (%)	
A generic drug product is not comparable to an innovator drug product in dosage form, strength, route of administration, quality, performance characteristics, and intended use	292^a^ (71.0)		92 (22.4)		27 (6.6)	
	S 137 (62.8)	P 155 (80.3)	S 67 (30.7)	P 25 (13.0)	S 14 (6.4)	P 13 (6.7)
A Drug Master File (DMF) is a submission to the Food and Drug Administration (FDA) that may be used to provide confidential detailed information about facilities, processes, or articles used in the manufacturing, processing, packaging, and storing of one or more human drugs.	222^a^ (54.0)		158 (38.4)		31 (7.5)	
	S 103 (47.2)	P 119 (61.7)	S 96 (44.0)	P 62 (32.1)	S 19 (8.7)	P 12 (6.2)
The Common Technical Document (CTD) is a set of specifications for the application dossier, for the registration of medicines	205^a^ (49.9)		189 (46.0)		17 (4.1)	
	S 98 (45.0)	P 107 (55.4)	S 112 (51.4)	P 77 (39.9)	S 8 (3.7)	P 9 (4.7)
Active Pharmaceutical Ingredient (API) is not related to drug substance	305^a^ (74.2)		89 (21.7)		17 (4.1)	
	S 150 (68.8)	P 155 (80.3)	S 61 (28.0)	P 28 (14.5)	S 7 (3.2)	P 10 (5.2)
Common Technical Document (CTD), is a format set by the ICH	182^a^ (44.3)		207 (50.4)		22 (5.4)	
	S 89 (40.8)	P 93 (48.2)	S 114 (52.3)	P 93 (48.2)	S 15 (6.9)	P 7 (3.6)
The regulatory agency in Jordan is the Jordan Food and Drug Administration (JFDA)	302^a^ (73.5)		89 (21.7)		20 (4.9)	
	S 143 (65.6)	P 159 (82.4)	S 63 (28.9)	P 26 (13.5)	S 12 (5.5)	P 8 (4.1)

^a^: Correct answer.

S: Students (n = 218).

P: Pharmacists (n = 193).

With regards to participants’ perception of RA and DR (Table 5), 38.9% agreed/strongly agreed that the schools of pharmacy in Jordan should introduce their students to RA, 74.4% agreed/strongly agreed that there is a lack of awareness about RA among pharmacists in Jordan, and 81.5% agreed/strongly agreed that more workshops, lectures, and training regarding RA and DR are required.

**Table 5:** Participants’ perception regarding RA and DR (n = 411).

Statement	Strongly agree n (%)		Agree n (%)		Neutral n (%)		Disagree n (%)		Strongly disagree n (%)	
The schools of pharmacy in Jordan should introduce their students to RA	95 (23.1)		65 (15.8)		135 (32.8)		85 (20.7)		31 (7.5)	
	S 51 (23.4)	P 44 (22.8)	S 40 (18.3)	P 25 (13.0)	S 86 (39.4)	P 49 (25.4)	S 31 (14.2)	P 54 (28.0)	S 10 (4.6)	P 21 (10.9)
The roles of regulatory RA are well-known by pharmacists in Jordan	82 (20.0)		79 (19.2)		134 (32.6)		86 (20.9)		30 (7.3)	
	S 47 (21.6)	P 35 (18.1)	S 51 (23.4)	P 28 (14.5)	S 77 (35.3)	P 57 (29.5)	S 34 (15.6)	P 52 (26.9)	S 9 (4.1)	P 21 (10.9)
There is a lack of awareness about RA among pharmacists in Jordan	180 (42.8)		130 (31.6)		82 (20.0)		16 (3.9)		3 (0.7)	
	S 84 (38.5)	P 96 (49.7)	S 70 (32.1)	P 60 (31.1)	S 52 (23.9)	P 30 (15.5)	S 11 (5.0)	P 5 (2.6)	S 1 (0.5)	P 2 (1.0)
More workshops, lectures, and training regarding RA and DR are required	249 (60.6)		86 (20.9)		63 (15.3)		12 (2.9)		1 (0.2)	
	S 117 (53.7)	P 132 (68.4)	S 52 (23.9)	P 34 (17.6)	S 39 (17.9)	P 24 (12.4)	S 9 (4.1)	P 3 (1.6)	S 1 (0.5)	P 0 (0.0)

S: Students (n = 218).

P: Pharmacists (n = 193).

According to the multiple linear regression analysis, none of the independent variables affected the goals’ knowledge score (Table 6). On the other hand, concerning roles’ knowledge score, pharmacist participants (p-value = 0.022) or those who previously attended a lecture related to RA and DR (p-value = 0.012) had significantly higher roles’ knowledge scores. Furthermore, previously attending a lecture related to RA and DR (p-value = ≤ 0.001) significantly influenced the general knowledge score (Table 6).

**Table 6:** Assessment of factors affecting knowledge scores among study participants (n = 411).

Parameter	Goals’ knowledge score				Roles’ knowledge score				General knowledge score			
	Beta	P-value^a^	Beta	P-value^b^	Beta	P-value^a^	Beta	P-value^b^	Beta	P-value^a^	Beta	P-value^b^
**Age**												
≤ 23 years old ≥ 24 years old	Ref. 0.073	0.164	-0.032	0.661	Ref. 0.153	0.003	0.025	0.728	Ref. 0.136	0.010	0.019	0.793
**Gender**												
Male Female	Ref. 0.063	0.206	0.064	0.230	Ref. 0.054	0.276	---	---	Ref. -0.025	0.608	---	---
**Pharmacists or pharmacy students?**												
Pharmacy students Pharmacists	Ref. 0.157	0.001	0.131	0.078	Ref. 0.220	≤0.001	0.168	**0.022** ^s^	Ref. 0.175	≤0.001	0.137	0.055
**Where did you study pharmacy?**												
Outside Jordan Jordan	Ref. -.011	0.818	---	---	Ref. -0.038	0.445	---	---	Ref. -0.018	0.719	---	---
**Private or Public university?**												
Private university Public (governmental) university	Ref. 0.035	0.474	---	---	Ref. 0.015	0.760	---	---	Ref. 0.025	0.620	---	---
**During your undergraduate studies, did you take** a **course or topic related to RA?**												
No Yes	Ref. 0.049	0.318	---	---	Ref. 0.046	0.357	---	---	Ref. 0.192	≤0.001	0.061	0311
**Did you attend any lectures or workshops on RA and DR?**												
No Yes	Ref. 0.123	0.012	0.102	0.056	Ref. 0.178	≤0.001	0.131	**0.012** ^s^	Ref. 0.290	≤0.001	0.228	≤**0.001**^s^

^a^: Using simple linear regression,

^b^: Using multiple linear regression,

^s^: Significant at 0.05 significance level.

## Discussion

The RA represents a fundamental component of present pharmaceutical industries to register pharmaceutical products in a universal and standardized manner [1]. The requirements for the registration of pharmaceutical products are harmonised in regulated countries by using an internationally acceptable format known as CTD established by the ICH [6]. All regulatory agencies worldwide adhere to this international format to register and approve drugs. The Jordan Food and Drug Administration (JFDA) is the regulatory agency in Jordan tasked with ensuring the safety and quality of pharmaceutical products [13,14], and has succeeded in implementing regulations as per international regulatory agencies’ guidance and requires the pharmaceutical company to fulfill the CTD format [15], since Jordan is among the regulated countries that commit to the ICH guidelines.

The current study, being the first to assess the knowledge and perceptions of RA and DR among pharmacists in Jordan, was designed to cover different levels of pharmacy including pharmacy students, who comprised half of the participants, with the other half being pharmacists. Interestingly, the majority of the participants knew the definition of RA (78.3%), and the definition of DR (77.1%). This illustrates good theoretical knowledge of the two subjects. Despite the fact that the majority of the participants were not exposed to such topics during their university studies (77.4%), or even attended workshops in the past (70.8%); they tried to gain information using several resources such as electronic websites and social media to keep up with this rapidly developing field of pharmacy. Additionally, the participant’s knowledge regarding the goals of RA showed that they are generally well-informed about these goals. Furthermore, their knowledge of the roles bound to RA professionals was shown to be higher than what was found among the pharmacists in India [16].

Interestingly, knowledge concerning the roles of RA professionals was higher among pharmacists and those who previously attended a lecture related to RA and DR. This reinforces the potential benefits of workshops and lectures and enhances the awareness and understanding of the field of RA and DR, which also showed a greater general knowledge score among the respondents.

Worth mentioning is that while the number of students aligns closely with the 18–23 age category, the regression analysis shows the significance of the correlation between the age categories and the total knowledge scores. The high percentage in the 18–23 age category ensures adequate representation for analysis, however, it does not necessarily lead to significant correlations, as these are influenced by other variables within the linear regression analysis, such as gender, being a pharmacist or student, university type, and previous exposure to related courses or workshops.

The PSUR, an important annual PV report submitted to health authorities, is used to ensure the safety of the authorized pharmaceutical product. Aligning with that, the health authority in Jordan, JFDA, established its PV system in 2001 and became a member of WHO in 2002 [17]. Moreover, in alignment with global regulations, an Adverse Drug Reaction (ADR) reporting system based on the WHO reporting system has been established to monitor and track any rising safety concerns related to the marketed pharmaceutical product and the adverse effects reported by healthcare providers, industry, and patients [18].

Since not all adverse effects can be anticipated during the clinical studies and prior to drug approval [19], post-marketing surveillance is essential to monitor and report any unexpected adverse effects after the drugs are launched in the market [20,21]. The PV definition was evaluated in the current study, and the results revealed that only 48.4% (n = 199) of the participants were aware of the definition which was better appreciated when compared to 8.7% among Jordanian pharmacists and pharmacy students back in 2018 [22], and 19.1% among pharmacists practicing in Yemen [23]. On the other hand, a better understanding of PV was reported among Saudi and Indian pharmacy and healthcare students (60.85%, and 86.74%, respectively) [16,24].

The CTD is a collection of documents that contain all the technical data of the pharmaceutical product as per the ICH. Regarding such an important part of RA and DR responsibilities [25], inconsistent knowledge about CTD was present. While about half of the participants were aware of the CTD definition, unfortunately, only a fifth knew that the CTD is divided into five modules. In the study conducted in India almost half of the participants correctly identified the number of CTD modules [16]. Furthermore, knowledge of the CTD modules’ specifics, particularly modules 2 and 3, revealed deficient awareness contrary to the study done in India [16].

Markedly, a great majority of the participants did not take any course related to RA during their university studies or even attended any RA workshops. This underscores the importance of introducing RA courses within pharmacy schools’ curricula in Jordan, in order to educate and raise the pharmacy students’ knowledge and awareness of regulatory agencies’ aims, rules, legislations, regulations, procedures, and policies. This would create a new career path for freshly graduated pharmacists in RA departments since the other ordinary paths such as community pharmacy and hospital roles were getting saturated due to the increasing number of pharmacy schools and graduated pharmacists each year in Jordan [14]. This would render the graduates capable of fulfilling the demands of the RA departments in the local pharmaceutical industry which has been experiencing rapid growth in recent years [13]. The inclusion of RA courses in faculties of pharmacy in Jordan is an indispensable requirement, as is the case in other countries around the world [26].

The participants believed that there is a lack of awareness about RA among pharmacists in Jordan and agreed on the importance of conducting and holding more workshops, lectures, and training in RA and DR. Moreover, consistent with the findings that previously attending RA workshops increased the participants’ knowledge of RA and DR, workshops and awareness lectures can be considered an effective measure to build a firm background and lay the general understanding necessary for every pharmacist. To undertake effective and sustainable measures, a collaborative scheme conjoining the JFDA, the faculties of pharmacy in Jordanian universities, the Jordan Pharmaceutical Association (JPA), and the pharmaceutical industry should take shape. Such interplay can install effective training workshops concerned with RA and DR capable of providing the local market with qualified and specialized pharmacists in the field.

### Strength and limitations

This work can be considered one of the first to comprehensively evaluate and shed light on the RA profession in pharmaceutical industries, which is considered among the new and very important fields of work and career path for pharmacists, especially new graduates. Nonetheless, the current study comes with some limitations. First, the convenience sampling technique adopted in the study may limit the generalizability of the study findings. Also, the size of the population, despite being adequate to perform the analysis, did not have a great representation of all the different groups within the study. Furthermore, the study’s analysis did not compare knowledge levels among pharmacists based on their specific roles or job types.

## Conclusion

The RA knowledge among pharmacy students and pharmacists in Jordan was shown to be inadequate. This study aims to elevate Jordanian pharmacists’ awareness, taking into account the duties of the regulatory bodies in Jordan collaboratively with pharmacy schools, by placing great effort on young pharmacists and pharmacy students. This can be achieved by implementing RA courses within the pharmacy curricula, holding workshops, and harnessing social media to reach out to all pharmacists in different settings. Future studies should consider exploring these differences to provide more tailored perceptions.

## Supporting information

S1 Questionnaire Study questionnaire. (DOCX)

S2 Data Supporting data. (CSV)
