# Supplementary material for: Drug regulatory affairs under focus: Knowledge and perceptions among pharmacists and pharmacy students
Source: PLoS One. 2025 Mar 27;20(3):e0320599. doi: 10.1371/journal.pone.0320599 (PMC11949326; doi:10.1371/journal.pone.0320599)
Supplement: S1 Questionnaire — (DOCX) [file pone.0320599.s001.docx]

**Knowledge and awareness of Regulatory affairs (RA) and Drug registration among pharmacists and Pharmacy Students**

**Informed Consent Form**

**Dear participant**

Researchers are carrying out a research project. This survey is aimed to address pharmacists` and pharmacy students` knowledge and awareness about Regulatory affairs career.

Your cooperation is highly appreciated and is indeed of high importance.

No personal information is required. Collected data will not be dealt with confidentially and with utmost privacy and will not be used outside the scope of the research.

Your participation in this research study is voluntary. You may choose not to participate. If you decide to participate in this research survey, you may withdraw at any time.

We would like to confirm that all information provided here will be kept confidential. All data will be stored in a password protected electronic format. To help protect your confidentiality, the surveys will not contain information that will personally identify you and will be used only for research purposes.

The procedure involves filing out an online survey that will take approximately 10-15 minutes. Your participation in completing this survey is highly appreciated.

**ELECTRONIC CONSENT**: Please select your choice below.

Clicking on the "agree" button below indicates that:

1. You have read the above information
2. You voluntarily agree to participate

If you do not wish to participate in the research study, please decline participation by clicking on the "disagree" button.

- Agree
- Disagree

**Knowledge and awareness of Regulatory affairs (RA) and Drug registration among pharmacists and Pharmacy Students**

**Part 1. Demographic Information**

Age: (in years)

- 18-23
- 24-30
- 31-40
- >40

Gender:

 Female  Male

Are you a pharmacist or a pharmacy student? (please choose the one that best fits you)

- Pharmacy student (first or second academic year)
- Pharmacy student (third, fourth or fifth academic year)
- Pharmacist

Which of the following describes your job?

- Academia
- Pharmacy
- Drug store
- Pharmacy Student

How long has it been since you got your pharmacy degree (BSc in pharmacy)?

- 1-3 years
- 4-10 years
- 11-20 years
- More than 20 years
- I am still a pharmacy student

Nationality

 Jordanian  Others (please specify)

Place of residence

 Amman  Other (please specify)

Where did you study pharmacy?

 Jordan  Other

Are you studying/ have you studied at public (governmental) or private universities?

 Public (governmental) University  Private University

During your undergraduate studies, did you take a course or topic related to regulatory affairs (RA)?

 Yes  No

**Part 2. Knowledge about regulatory affairs and drug registration**

---------------------------is the process of submitting an application to regulatory authorities seeking approval to market and distribute a drug product.

- Drug registration
- Drug regulatory affairs

------------------ is the overall management of regulatory activities related to drug development, registration, and post-marketing surveillance to ensure that all regulatory requirements are met throughout the drug development process

- Drug registration
- Drug regulatory affairs

What are the goals of Regulatory Affairs Professionals

- Protection of human health
- Ensuring safety, efficacy and quality of drugs
- Ensuring appropriateness and accuracy of product information

What are the Roles of Regulatory Affairs professionals?

- Act as a liaison with regulatory agencies
- Preparation of organized and scientifically valid new drug application and drug master file
- Ensure adherence and compliance with all the applicable cGMP, ICH, GCP, GLP guidelines, regulations and laws
- Providing expertise and regulatory intelligence in translating regulatory requirements into practical workable plans
- Advising the companies on regulatory aspects and climate that would affect their proposed activities

What is the responsibility of RA personnel?

- To analyze the content of the active ingredient in the formulation
- Work with regulatory authorities to get the approval for drugس
- To undertake stability studies of the drug products
- To supervise the production of the formulation
- I don`t know

Identify the activity related to Pharmacovigilance

- Deals with animal studies for pharmaceutical products.
- Deals with export of drug product.
- Deals with detection, monitoring and prevention of adverse effects with pharmaceutical product.
- Deals with manufacturing and packaging of pharmaceutical products
- I don`t know

The initiation of ICH took place with representatives of regulatory agencies of --------- to discuss the wider implications and terms of reference.

- Japan, Australia, US
- US, Europe, India
- US, Europe, Japan
- Europe, Australia, US
- I don’t know

Common Technical Document (CTD) is divided into ---------- modules

- 2
- 3
- 4
- 5
- 6
- I don`t know

CTD module 2 is related with -------

- Administrative information and prescribing information
- All CTD summaries
- Quality of pharmaceutical products
- Nonclinical Study
- I don`t know

CTD module 3 is related with -------

- Administrative information and prescribing information
- All CTD summaries
- Quality of pharmaceutical product
- Non clinical Study
- Clinical study
- I don`t know

Your source of information about Regulatory Affairs

- Electronic website
- Academic institution
- Pharmaceutical companies/Industries
- Social media
- Friends

**Part 3. Knowledge about Regulatory Affairs and Drug registration with Yes or No answer**

- A generic drug product is the one that is comparable to an innovator drug product in dosage form, strength, route of administration, quality, performance characteristics and intended use.
- A Drug Master File (DMF) is a submission to the Food and Drug Administration (FDA) that may be used to provide confidential detailed information about facilities, processes, or articles used in the manufacturing, processing, packaging, and storing of one or more human drugs.
- The Common Technical Document (CTD) is a set of specification for application dossier, for the registration of Medicines
- Active pharmaceutical ingredient (API) is related to drug substance
- Common Technical Document (CTD), is a format set by the ICH
- The regulatory agency in Jordan is JFDA

**Part 4. Perception of participants about RA and drug registration**

From the following statements please select your level of agreement

| **Statements** | Strongly agree | Agree | Neutral | Disagree | Strongly disagree |
| --- | --- | --- | --- | --- | --- |
| The Schools of pharmacy in Jordan introduce their students to RA | **** | **** | **** | **** | **** |
| The roles of RA professionals are well Known by pharmacists in Jordan | **** | **** | **** | **** | **** |
| There is lack of awareness about RA among pharmacists in Jordan | **** | **** | **** | **** | **** |
| More workshops, lectures and training regarding RA and drug registration are required | **** | **** | **** | **** | **** |
